# Chromosomes in a genome-wise order: evidence for metaphase architecture

**DOI:** 10.1186/s13039-016-0243-y

**Published:** 2016-04-27

**Authors:** Anja Weise, Samarth Bhatt, Katja Piaszinski, Nadezda Kosyakova, Xiaobo Fan, Annelore Altendorf-Hofmann, Alongklod Tanomtong, Arunrat Chaveerach, Marcelo Bello de Cioffi, Edivaldo de Oliveira, Joachim-U. Walther, Thomas Liehr, Jyoti P. Chaudhuri

**Affiliations:** Institute of Human Genetics, Jena University Hospital, Postfach, 07740, Jena, Germany; Department of General, Visceral und Vascular Surgery, Jena University Hospital, Kochstr. 2, Jena, 07743 Germany; Department of Biology, Faculty of Science, Khon Kaen University, 123 Moo 16 Mittapap Rd, Khon Kaen, Muang District 40002 Thailand; Departamento de Genética e Evolução, Universidade Federal de São Carlos, São Carlos, SP Brazil; Instituto Evandro Chagas, Seção de Meio Ambiente, Laboratório de Cultura de Tecidos e Citogenética, Ananindeua, PA Brazil; Kinderklinik, Ludwig Maximillians Universität, 80337 Munich, Germany

**Keywords:** Genome architecture, Parental origin, Haploid grouping, Chromosomes, Metaphase

## Abstract

**Background:**

One fundamental finding of the last decade is that, besides the primary DNA sequence information there are several epigenetic “information-layers” like DNA-and histone modifications, chromatin packaging and, last but not least, the position of genes in the nucleus.

**Results:**

We postulate that the functional genomic architecture is not restricted to the interphase of the cell cycle but can also be observed in the metaphase stage, when chromosomes are most condensed and microscopically visible. If so, it offers the unique opportunity to directly analyze the functional aspects of genomic architecture in different cells, species and diseases. Another aspect not directly accessible by molecular techniques is the genome merged from two different haploid parental genomes represented by the homologous chromosome sets. Our results show that there is not only a well-known and defined nuclear architecture in interphase but also in metaphase leading to a bilateral organization of the two haploid sets of chromosomes. Moreover, evidence is provided for the parental origin of the haploid grouping.

**Conclusions:**

From our findings we postulate an additional epigenetic information layer within the genome including the organization of homologous chromosomes and their parental origin which may now substantially change the landscape of genetics.

**Electronic supplementary material:**

The online version of this article (doi:10.1186/s13039-016-0243-y) contains supplementary material, which is available to authorized users.

## Background

Recent studies showed that epigenetic information e.g. non-coding RNAs, DNA methylation or histone modifications are key regulators of gene expression (summarized in [1]). Besides there is another “layer” of epigenetic information on the level of higher order chromatin organization, which became more and more into the focus due to application of high resolution chromosome conformation capture assays (e.g. [2]). Nevertheless, these methods are neither able to distinguish between homologous chromosomes nor to delineate their parental origin. As apparent from chromosome territory studies by fluorescence in situ hybridization (FISH) there is a chromosome positional code which seems to be cell type and specific for time of development (e.g. [3, 4]). However, standard FISH-methods do not register the behavior of homologous chromosomes as well as their organization with respect to the parental origin, which we postulate here as an additional epigenetic “information layer”.

A non-random distribution of chromosomes was suggested already in the early days of human cytogenetics [5, 6]; however, majority of cytogeneticists commonly accept that chromosomes in a metaphase spread are generally arranged completely haphazardly. Based on the observation of the bilaterally symmetric distribution of DNA and chromosome specific FISH signals in leukocytes, we demonstrated in a series of publications [7–13] a genome-wise organization of chromosomes in human and murine cells. In other words, the parental haploid chromosome sets of diploid cells are well arranged within the nucleus and also within the metaphase stage, when DNA is most condensed and appears as microscopically visible chromosomes. Previously Gläss [14, 15] observed the segregation of parental haploid chromosome sets in regenerating liver cells of rats; also Pera [16] presented a hexaploid metaphase spread in vole cells, where the six sets of chromosomes were apparently lying within distinct haploid domains. Additionally, in insects and plants there are clear examples of separated parental genomes in the nuclei [17, 18].

As a rule, rather than an exception, we found this genome-wise haploid order of chromosomes in a variety of samples from different human tissues; in different species of macaque monkeys; in mice (*Mus musculus*); in aberrant human karyotypes with triploidy, tetraploidy, uniparental disomy (UPD); in human blood samples subjected to pod-FISH (parental origin determination FISH) [19] and samples with small supernumerary marker chromosomes (sSMC) [20]. The detailed analysis of three clinical cases with sSMC and UPD shed light on the functional role of this more general genome-wise order.

Our results show that there is not only a defined nuclear architecture in interphase but also in metaphase allowing bilateral organization of the two haploid sets of chromosomes. Moreover, evidence is provided for the parental origin of the haploid groupings.

## Results

The homologous chromosome organization and their haploid grouping was investigated in normal human metaphase spreads of a family trio and on aberrant metaphases of different clinical cases and tissues to get hints on the functional relevance of the previously observed higher order organization of metaphases. Haploid grouping was done by drawing a symmetry line which separates the two haploid genomes in the metaphase. To demonstrate the general principle of haploid grouping in the metaphase stage of the cell cycle we explored additionally different primate species and murine samples.

### Human family trio analyzed with pod-FISH

**Fig. 1:**
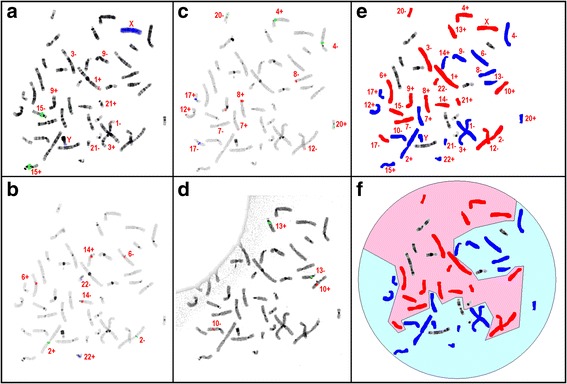
Example of sequential hybridizations of informative pod-FISH probe sets on a proband metaphase spread (**a**-**d**). According to the parental code from Fig. 1 the chromosomes of the proband were labeled with blue for paternal origin and red for maternal origin (**e**). According to the parental origin a bilateral symmetry and a grouping of the maternal and the paternal chromosomes could be visualized in the proband metaphase (**f**)

**Fig. 2:**
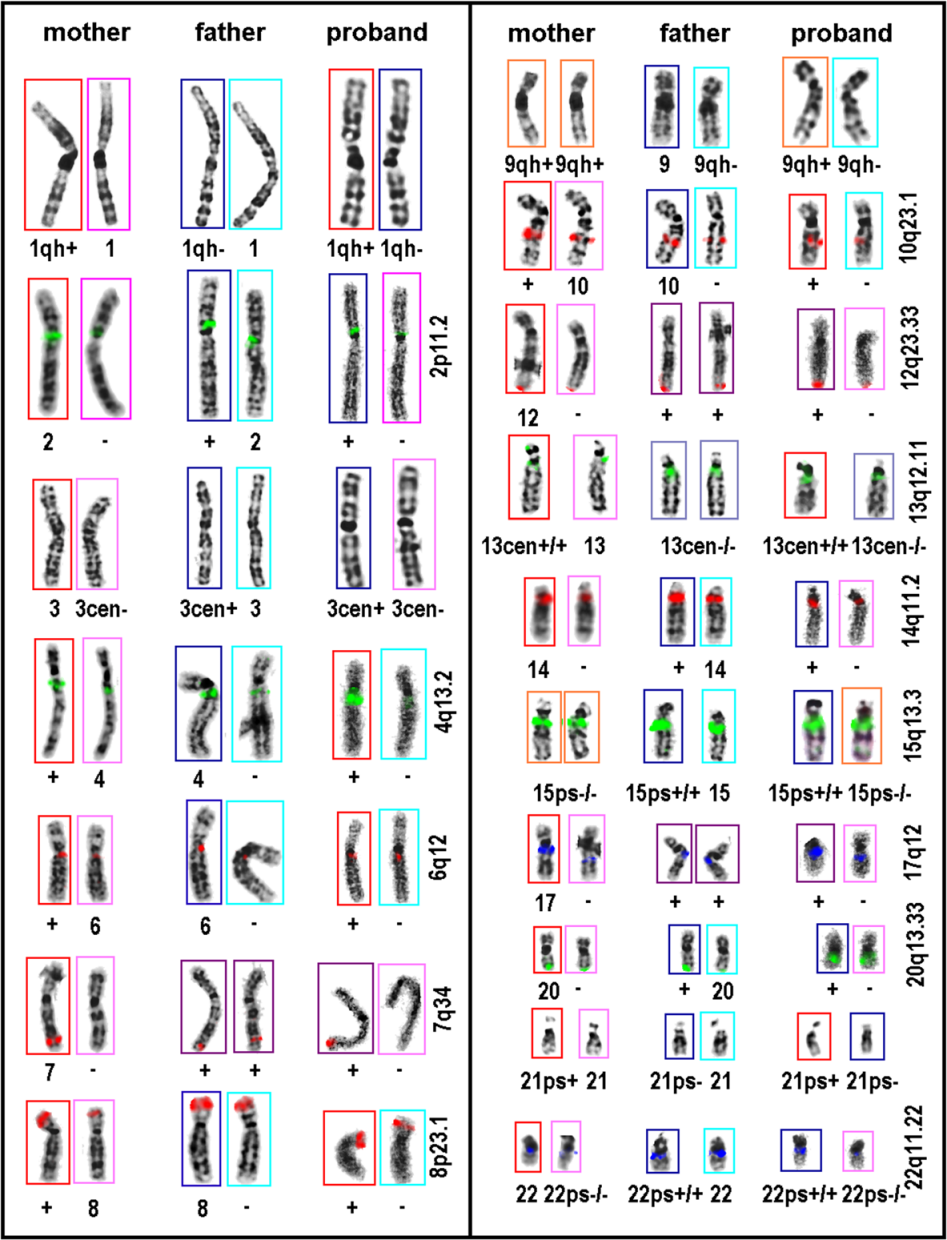
Decoding parental origin for the listed chromosomes by pod-FISH probes of corresponding cytogenetic polymorphisms

In order to analyze the organization of homologous chromosomes to each other and also with respect to their parental origin we applied pod-FISH on a family trio. Polymorphic FISH-probes, due to copy number variations which allow the distinction of homologous chromosomes, were used to draw conclusions on the parental origin of single homologous chromosomes in the child of the trio 170 metaphase spreads from peripheral blood of a normal male proband (child of a family trio) were analyzed for perfect bilateral symmetry of haploid chromosome sets, found in 51 from 170 cells (~30 %, examples are given in Additional file 1: Figure S1). 26 of these metaphases were analyzed by pod-FISH with sequential hybridizations (Fig. 1). In parallel, metaphase spreads of each parent were also analyzed by pod-FISH to determine the parental origin of 17 autosomes, X- (and Y) chromosomes in the proband (Fig. 2). Besides the bilateral symmetry of haploid chromosome sets, a clear parental-wise grouping of the two haplosets was found in 3 out of 26 metaphase spreads analyzed (11.5 %) (exemplified in Fig. 1).

### Bilateral symmetry line often reflects a mirror line dividing pairs of homologous chromosomes next to each other

**Fig. 3:**
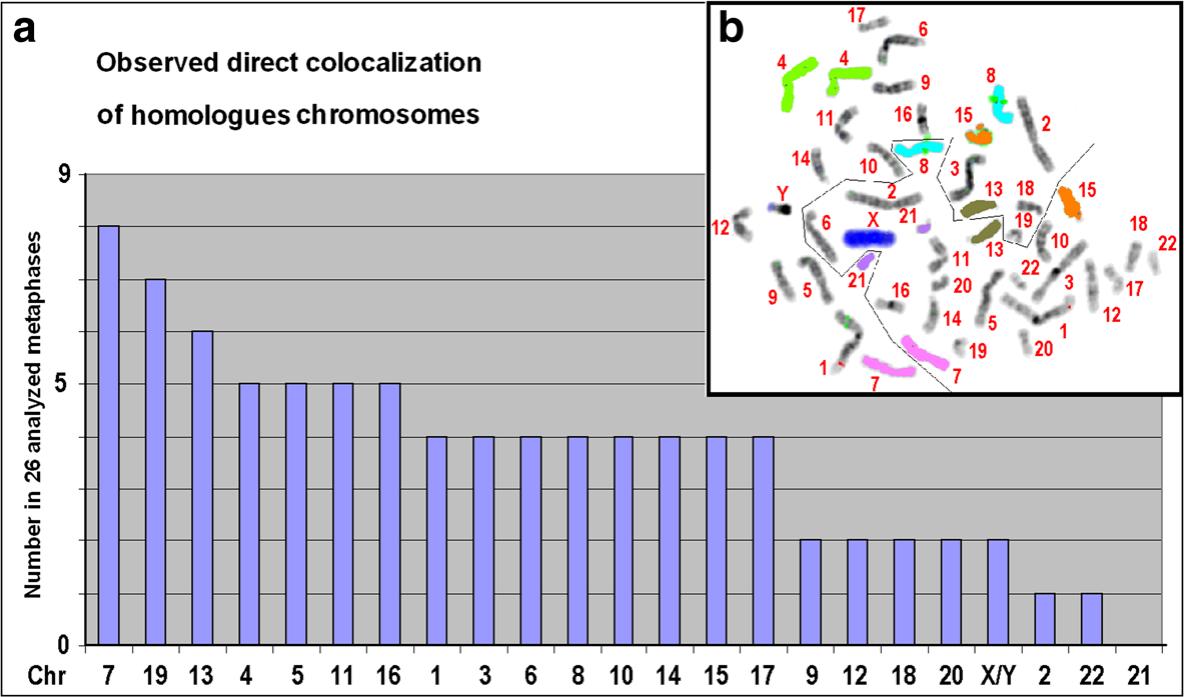
**a** Observed direct co-localization of homologous chromosomes in 26 metaphase spreads of a normal male individual in order of observed frequencies per chromosome. **b** Example of one metaphase where chromosomes 4, 7, 8, 13, 15 and 21 labeled in different colors show direct co-localization within the metaphase plate. Further examples are given in Additional file 3: Figure S3

The 26 metaphase spreads examined by pod-FISH were also analyzed for co-localization of homologous chromosomes, previously described as somatic pairing (Fig. 3). 85 instances of direct co-localizations were counted with a mean frequency of 3.3 per metaphase spread. The frequency per chromosome is not constant and favors predominantly chromosome 7 (31 % of cells), 19 (27 % of cells) and 13 (23 % of cells). On the other hand, a direct co-localization of both chromosomes 21 was never observed and chromosomes 2 and 22 were co-localized only in 1 out of 26 metaphase spreads (4 %).

### Clinical case with aberrant bone marrow karyotype

Twenty metaphase spreads from a male patient diagnosed with acute myelogenous leukemia (AML) and trisomy 8 mosaicism in bone marrow were evaluated (47,XY,+8[15]/46,XY[5]). After grouping of haplosets the additional chromosome 8 was analyzed for affiliation to maternal or paternal haplogroup. Eleven trisomy 8 metaphases showed a location within the paternal haploset and four were assigned to the maternal haploset. Furthermore, 5 out of 15 trisomy 8 metaphases (33 %) exhibited a “next to next location” of two chromosomes 8 (Additional file 2: Figure S2).

### Clinical cases with aberrant amniotic fluid and fibroblasts

**Fig. 4:**
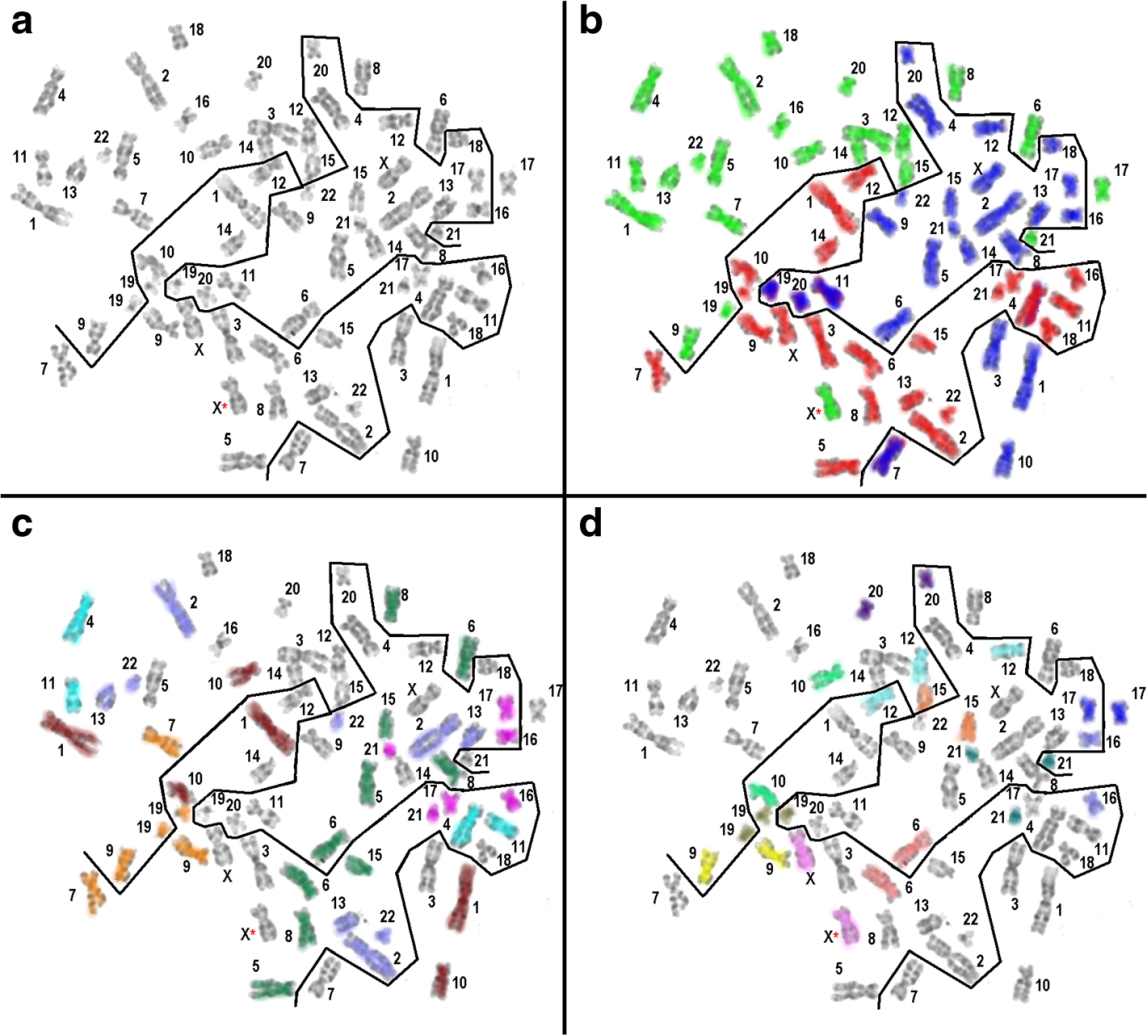
Metaphase example from a triploid amniotic fluid after in situ preparation demonstrating a genome-wise sorting of the three haploid chromosome sets. (**a**) inverted DAPI, (**b**) each haploid set labeled in blue, red and green respectively, (**c**) chromosome grouping and (**d**) close location of homologous chromosomes next to the symmetry line

Homologous chromosome haploset grouping, as well as the grouping of single chromosome combinations in a mirror-image manner and the location of homologous chromosomes along the symmetry line was also observed in tri- and tetraploid human metaphases from abortion fibroblasts and amniotic fluid cells after in situ preparation (exemplified in Fig. 4).

### Clinical cases with sSMC

Twenty metaphase spreads were evaluated for each case outlined below, by drawing a line separating two haploid chromosome sets and subsequent analysis of the additional sSMC location and, in case of UPD analysis, of the affected homologous chromosomes.

### Case 1: mos 47,XY,+min(14)(pter→ q11.1)dn, upd(14)mat heterodisomy/46,XY,upd(14)mat heterodisomy

A male patient with de novo sSMC(14) in 50 % of his cells and maternal heterodisomy 14 presented at the age of 31 with short stature and adiposity.

Bilateral symmetry line analysis showed that the sSMC was present in 11/20 metaphases and located in 6/11 on the maternal/X chromosomal haploset. Additionally, the marker chromosome was found in 7/11 metaphases next to the symmetry line, which allows also an alternative drawing of the latter (Additional file 3: Figure S3A).

Chromosomes 14 presenting maternal heterodisomy were tested with a centromere 14/21 specific probe for heteromorphisms and the chromosome 14 with a stronger centromeric signal turned out to be located in 14/20 on the paternal and in 6/20 on the maternal site (Additional file 3: Figure S3A). In 5/20 metaphases, both homologous chromosomes, and in 15/20 at least one chromosome 14 was located next to the symmetry line.

### Case 2: mos 47,XY,+min(7)(:p13 → p11.1:)dn,upd(7)mat isodisomy/46,XY,upd(7)mat isodisomy

A four month old boy with a de novo sSMC(7) in 8 % of the cells and additional maternal isodisomy of the homologous chromosomes 7 showed dystrophy, developmental delay and abnormal ears.

Only 1/20 metaphases showed the sSMC located on the paternal/Y chromosomal haploset, but next to the symmetry line. Both isodisomic chromosomes 7 presented in 20/20 metaphases a clear grouping to one of the haplosets and in 14/20 metaphases both chromosomes 7 were located next to the symmetry line (Additional file 3: Figure S3B).

### Case 3: 47,XY,+inv dup(22)(q11.2)mat

An additional inverted duplicated sSMC(22) inherited from the phenotypically normal mother was present in 100 % of the amniocytes in a male fetus.

In all 20 metaphase spreads the sSMC was present and located in 12/20 on the maternal/X chromosomal and in 8/20 on the paternal/Y chromosomal haploset. Furthermore, in 12/20 metaphases the sSMC was located next to the symmetry line. In 17/20 metaphases both haplosets included one of the homologous chromosomes 22 and in 10/20 metaphases both homologous were located next to the symmetry line (Additional file 3: Figure S3C).

### Primate metaphases

Ten different primary primate samples, detailed in Additional file 4: Table S1, were hybridized with human M-FISH probe sets and at least 5 metaphases were evaluated each, for bilateral symmetry, location of homologous chromosomes next to the symmetry line and chromosome grouping. With the exception of only a single chromosome, which escaped the haplotype grouping and was preferentially located on the outer area of the metaphase, all 50 metaphases analyzed from the 10 different primate species showed a bilateral grouping of chromosomes in haplosets (examples are given in Additional file 5: Figure S4).

Additionally, the metaphases showed also a chromosome grouping and a close location of homologous chromosomes next to the symmetry line as observed in human metaphases (Figs. 3 and 4).

### Murine metaphases

Twelve mouse metaphase spreads from primary spleen preparation were analyzed with a mouse specific M-FISH probe set and evaluated for bilateral symmetry, location of chromosomes next to the symmetry line and chromosome grouping.

The metaphases showed a grouping of the chromosome sets and a close location of homologous chromosomes next to the symmetry line (Additional file 6: Figure S5) as seen in the other samples before.

## Discussion

### The influence of preparation methods on metaphase architecture

Even though human chromosomes have been prepared uncountable times using the air drying approach for over 50 years, structure and process of chromosome spreading were not understood for a long time. Recent studies revealed that fixed lymphocytes at the metaphase stage spread after being attached to the slide surface [21] rather than “burst” their metaphase plates as suggested for years. This surprisingly slow process is humidity-dependent [22] and is driven by the evaporation of Carnoy’s fixative. First methanol evaporates, followed by acetic acid. As acetic acid is hydrophilic, water is acquired from the atmosphere and the chromosomes elongate due to a stretching or swelling process [21, 23].

In earlier studies by methylation staining of mammalian spermatocytes as well as early embryonic cells the parental order and genomic separation of parental chromosome sets were detected [24]. While this parentally derived methylation pattern disappeared after the four-cell stage, the chromosomes still remained aligned during interphase (chromosome territories) and even during metaphase as suggested by us previously [7, 10–13].

Although the standard preparations were used to arrest metaphase and treat them with hypotonic shock, here large numbers of metaphase spreads were detected with clear genome-wise separation of chromosomes. Additionally, in pod-FISH sampling a distinct sorting of the maternal and paternal chromosome sets was possible, except for single chromosomes lying astray. Also human metaphase spreads from bone marrow prepared without colcemid and showed the same results. Thus, an effect of colcemid on metaphase architecture can be excluded. This was also supported by 2D and 3D chromosome territory studies with different colcemid exposure times or without colcemid treatment [25]. Furthermore, human metaphases from abortion material prepared directly on slides by in situ culture showed in principle haploid sorting of even triploid chromosome sets, as well (some single chromosomes from metaphase edge escaped due to physical forces during preparation, partially masking metaphase architecture).

### Interphase architecture

In the end of the 19^th^ century Rabl [26] and Boveri [27] suggested the occurrence of an organized domain structure in the nucleus, which are nowadays known as chromosome territories (CTs) [28]. These nuclear CTs show a functional character reflected by spatial, temporal and cell type specific organization [29].

Several models exist to explain this organization:

1. Chromosomes have a centromere-telomere orientation which is stable during anaphase and cytokinesis, with the two chromosome arms lying next to each other and the centromeres and telomeres located at opposite poles of the nucleus [26, 30]. Nevertheless, this “Rabl configuration” is said to be rarely observed in mammalian cells [29].
2. The radial model predicts central location of gene rich chromosomes (like in *Homo sapiens* = HSA #1, #17, #19, and #22) in contrast to gene poor chromosomes like HAS #4, #5, #8, #13 or #18, independent of their size [31]. Cytogenetic preparations in pre-colchicine era often had metaphase plates with smaller chromosomes located centrally.
3. Nagele [32] suggests a relative chromosome domain positioning model also for the homologous chromosomes, predicting a preferred positional relationship to each other in the interphase and prometaphase of the cell cycle. Although not stated, this model already suggests the organization of chromosomes in two haploid sets as indicated by us [7].
4. The last model also predicts the presence of a certain architecture of CTs in both interphase and metaphase in the way of a haploid grouping, which beyond human samples [7–13] was also found in plant louse cells [17], rat liver cells [14, 15] and in hexaploid vole cells [16].

### Metaphase architecture and ploidy-wise bilateral sorting of chromosomes

In the present study this genome-wise haploid order of chromosomes was found as a rule, rather than an exception. From a variety of human samples, which include numerically and structurally abnormal human karyotypes and also in those derived from other species, including primates and mice the following implications can be assigned as a general model for metaphase architecture:

- Metaphase spreads show a more or less round shape similar to interphase nuclei, which in 3D represents a symmetrical distribution of the chromosomal DNA (Additional file 7: Figure S6). Interestingly, a round shape and equal DNA distribution can also be observed in so called “rosette” orientation of metaphase spreads, known classically as metaphase plates, where chromosome haplosets are arranged in a mirror wise order of homologous chromosomes [7, 11, 13]. This has already been observed in vivo and documented by the founder of cytogenetics Walther Flemming in salamander larva (Additional file 8: Figure S7) and quite recently by our group [13].
- The bilateral symmetry line often reflects a mirror line dividing two homologous chromosomes located next to each other (Figs. 3, 4; Additional file 6: Figure S5).
- This bilateral symmetry line is not always straight, since it can appear as a half circle where one haploset is surrounding the other (Additional file 1: Figure S1, Additional file 2: Figure S2, Additional file 3: Figure S3, Additional file 5: Figure S4). This might be due to metaphase chromosomes aligning in the equatorial plane of the cell (i.e. a 2D plane). Depending on the preparation and the angle of the 3D cell being fixed in 2D to the slide’s surface, the ideal form of a rosette shaped metaphase spread is “lost” and the symmetry line can appear in different shapes.
- Some chromosomes escaping the bilateral symmetry are often located in the outer area of the metaphase spreads, which might be due to physical shearing forces during preparation (Additional file 5: Figure S4).
- These features of metaphase architecture are independent of the origin of material, cultivation, preparation and species, reflecting a general model for the metaphase stage of the cell cycle (Figs. 1, 2, 3, 4, Additional file 1: Figure S1, Additional file 2: Figure S2, Additional file 3: Figure S3, Additional file 4: Table S1, Additional file 5: Figure S4, Additional file 6: Figure S5, Additional file 7: Figure S6, Additional file 8: Figure S7).

### Connecting interphase with metaphase architecture

Chromosomal neighborhoods seem to be dynamic within tissues and dependent on the cell cycle stage [3, 33, 34]. However, there are observations that frequent constitutional and acquired chromosomal translocation partners are located in close proximity in the nucleus and therefore more likely to interact [4, 33, 35]. Due to the molecular analysis used in Hi-C techniques, the interaction of homologous chromosomes within the two haplosets and between the two haplosets could not be analyzed. In our metaphase-directed analysis of chromosome neighborhoods we showed that homologous chromosomes are often direct neighbors separated by the bilateral symmetry line (Figs. 3, 4 and Additional file 6: Figure S5). Furthermore, certain homologous chromosomes preferentially tend to be located next to each other (chromosomes HSA #7, #19, #13, #4, #5, #11 and #16) at least in the detailed analysis of human peripheral blood cells (Fig. 3). Additionally, groups of chromosomes in a mirror-image manner, highlighting the preferred symmetry of DNA content and organization in all cell cycle stages, could be observed (Fig. 4, Additional file 6: Figure S5, Additional file 7: Figure S6 and Additional file 8: Figure S7).

One functional explanation for homologous neighborhood and mirror-image organization of chromosomes would be the advantage to minimizing connection costs in genetic networks, which is also discussed even for haploid stages in sperm that show a functional organization of genes expressed in the same tissue of an individual [36].

### Parental origin of haploid sets of chromosomes

Despite the extensive investigation of metaphases of peripheral blood lymphocytes in the past, so far no approaches were available to label the chromosomes in a parental origin specific manner. Here we applied pod-FISH to study the organization of the human nucleus on metaphase-spreads.

pod-FISH has already been successfully used to identify the parental origin of individual derivative chromosomes, such as the characterization of chimerism, derivative chromosomes and uniparental disomy 15 [19, 37–41]. For the first time we applied pod-FISH to analyze the architecture and the distribution of parental chromosomes on whole metaphase spreads. In a first step, 170 lymphocyte metaphase spreads of a healthy proband were karyotyped and a bilateral distribution of the homologous chromosome pairs, as described above, was found. The statistical probability to find such sorting by chance is 1 : $$ \left(\begin{array}{c}\hfill 23\hfill \\ {}\hfill 23\hfill \end{array}\right) $$ or 1 : 8,388,607. Then, 26 of these metaphases with a more or less perfect bilateral symmetry were subjected to sequential pod-FISH hybridizations and parental chromosome analysis (Figs. 1, 2 and Additional file 1: Figure S1). At least three of these also showed a clear parental grouping of the homologous chromosomes as shown in Fig. 1 which has a statistical chance of 1 : $\frac{46!}{23!\left(46-23\right)!}$ or 1 : 8,233,430,727,600. In other words, if this sorting would be by chance only 10–12 cells of the whole body would show such a parental grouping.

### Is bilateral order more important than the parental origin?

In order to address the functional consequences of parental grouping, we analyzed clinical cases with additional marker chromosomes with known parental origin. The additional chromosome in case 1 and 3 showed a random distribution but the marker had a higher frequency than firstly expected, being located next to the symmetry line also allowing an alternative drawing of the line or a central location within the metaphase in the sense of equal symmetrical distribution of DNA. Although the homologous of the marker chromosomes in case 1 and 2 presented a UPD, reflecting only one parental origin, in both cases the uniparental origin was “ignored” and the homologous chromosomes were located in one of the two haplosets each. This may highlight that, for the metaphase architecture maintaining an equal DNA distribution is more important than the location of single chromosomes according to their parental origin.

### Facilitating chromosome haplogrouping by centrioles

From a set of elegant experiments on chromosomal distribution in interspecies in vitro hybrid cells Teplitz [42] concluded that “in normal cells a mechanism (distribution control) strictly regulates movement of *a haploid set of mitotic chromosomes* into daughter cells upon cell division.”

The two separate groupings of the two parental sets of chromosomes are most likely achieved by the two centrioles in the centrosome of a diploid cell. Chromosomes of each haploset are presumably tethered to one of the two centrioles [10, 13]. The relation of centrioles to two halves of bilaterally lobulated nuclei of neutrophils support this hypothesis, as demonstrated using serial electron microscopic (EM) sections and cinematographic records [43–45]. Also Lettré and Lettré [45] reported that chromosomes, spindle fibres and centrioles form a permanent structure invisible during interphase. This observation was further supported by EM studies [46]. Krioutchkova and Onishchenko [47] claimed that the number of centrioles is exactly equal to the haplosets of chromosomes in a cell. At the same time, the only centriole in a fertilized egg is provided by the sperm [48]. The paternal chromosomes appear to be bound to this centriole, which initiates the creation of a second centriole to take care of the maternal chromosomes. This step is essentially crucial to achieve the order of separation of the parental haplosets of chromosomes and to maintain it thereafter both in interphase and metaphase, i.e. during the cell cycle [10]. The maintenance of this order is also supported by the lamin-sheath that covers all of the telophase chromosomes together, forming a ring or string which subsequently gives rise to a spheroid nucleus or folds into segments to form a polymorphic nucleus [13].

We also reported that this order of genome-wise grouping in human blood cells is expressed in three distinct forms: (1) the two parental genomes side by side; (2) one genome surrounded by the other; and (3) the members of the homologous chromosomes oriented opposite to each other [13]. Following our present podFISH analysis we have observed that the homologous chromosomes were often next to each other divided by the line of separation of the two parental genomes (Fig. 3). This feature in the 2D projection may mean that these members of homologous are nevertheless widely apart (i.e., diametrically opposite each other) on the Z-axis of a 3D configuration. The 3-D analyses of murine and human interphase nuclei, revealing that the average distance between the homologous are larger than that between the heterologous, support this feature [49, 50].

Observations of loss of maternal chromosome 11 [51] and especially genome-wide loss of maternal alleles in Wilms’ tumors [52], behavior of the three haplosets during gametogenesis in a bisexually reproducing triploid vertebrate [53] and the biphasic distribution of chromatin condensations of the two parental genomes [11] suggest that chromosomes are handled and/or addressed ploidy-wise. Maternal and paternal genomes may act alternately by opportunity or availability or necessity (cf. a mixed double tennis match). By necessity, the parental genomes do participate in harmonious cooperation, when, for example, the chromosomes bearing nucleolus-organizing-regions come together to form a nucleolus, even though they may remain tethered to their respective centrioles.

## Conclusion

In summary, we found a bilateral symmetry of metaphases leading to haploid sorting of homologous chromosomes and evidence of a parental grouping of these haploid sets. This indicates i) a higher order of chromosomal topography in the cell which is caused by the parental origin of homologous chromosomes, and we hypothesize that ii) this higher order is not limited to metaphase chromosomes but also represents an inherent feature of the interphase nuclei, iii) the cell distinguishes homologous chromosomes by the parental origin and iv) besides the horizontal sorting (equatorial plane) of chromosomes during metaphase, there is a vertical sorting by parental origin.

In the last decade nuclear architecture was recognized as an independent, emerging mechanism orchestrating gene expression (reviewed in [39]. The observation that homologous chromosomes - depending on their parental origin - have a defined position in the interphase nuclei as well as in the metaphase strengthens this concept and adds the parental origin information as an additional “epigenetic layer”. Architectural changes are priming events that happen before subsequent changes in gene expression and might therefore serve as future diagnostic and therapy markers e.g. in malignancys. which are well known as being associated with genetic instability and may very much be initiated by any loss of large scale chromatin order [54].

One can only speculate regarding impact and biological significance of this genome-wise order of chromosomes, however, it might be one additional mechanism leading to monoallelic expression of autosomal genes (summarized in [55]) and therefore contribute to normal phenotypic variation as well as to variable expressivity and incomplete penetrance of genetic diseases. Similarly, we may have to redefine the terms “Comparative Genomic Hybridization (CGH)” or “Loss of Heterozygosity (LOH)” by specifying the involvement of the maternal and/or paternal genomes in future or retrospective genotype phenotype correlations.

## Methods

Informed consent was obtained from all individual participants included in the study; the study was approved by the Ethics Committee of Friedrich Schiller Jena University Hospital (internal code 1457-12/04).

### Human and primate metaphases from peripheral blood culture and Epstein Barr virus (EBV) transformed permanent cell lines

Chromosome preparations were performed according to standard techniques [56]. An aliquot of heparinized peripheral blood (family trio and primate samples) or of frozen permanent cell lines transformed by the EBV was added to the cell culture medium, mixed with 10-20 % fetal calf serum, penicillin/streptomycin (10 mg/ml final conc.) and phytohemagglutinin (for primary blood cells, according to manufacture instruction). After 72 h of incubation at 37 °C/5 % CO_2,_ the cells were harvested. Thirty minutes before harvesting, colcemid (diacetylmethylcolchicine, 0.1 μg/ml final conc.) was added to arrest the cells at metaphase stage. The “air-drying method” of chromosome preparation [57] included hypotonic treatment with 0.075 M KCl for 20 min, a fixation step and several washing steps using Carnoy’s fixative (methanol/glacial acetic acid 3:1); finally, the suspension was dropped onto the slide’s surface.

Primate chromosome preparations (Additional file 4: Table S1) were provided by the coauthors from Thailand and Brazil.

Permanent EBV transformed cell lines from the clinical cases mos 47,XY,+min(7)(:p13 → p11.1:),upd(7)mat isodisomy/46,XY,upd(7)mat isodisomy (EKF-#7-p12/1-m) and 46,XY,upd(14)mat heterodisomy (EKF-#14-q11/1-m) were kindly provided by the Else Kröner-Fresenius (EFK) – cellbank (Institute of Human Genetics, Jena, Germany, http://ssmc-tl.com/ekf-cellbank.html).

### Human metaphases from abortion material and amniotic fluid

Chromosome preparations from abortion material and amniotic fluid material were performed according to standard techniques [56]. The primary abortion material was mechanically minced and covered with full medium mixed with penicillin/streptomycin and l-glutamate in a culture flask for 10–14 days at 37 °C/5 % CO_2_. Cells were disassociated by trypsin or in the case of amniotic fluid; a sedimented aliquote was transferred to a quadriperm plate on sterile glass slides with full medium for in situ culture for 7–10 days at 37 °C/5 % CO_2_. Ninety minutes before harvesting the cells colcemid (0.1 μg/ml final conc.) was added followed by hypotonic treatment (1.5 mM MgCl_2_, 0. 1 % sodium citrate and 150 U hyalorunidase) for 10–14 min, a fixation step and several washing steps using Carnoy’s fixative and final drying on the slide.

### Human metaphases from bone marrow material

Chromosome preparations from heparinized bone marrow were performed according to standard techniques [56]. An aliquot of bone marrow was added to the cell culture medium, mixed with 10–20 % fetal calf serum and penicillin/streptomycin. After 24 h of incubation at 37 °C/5 % CO_2_ the cells were harvested without colcemid. Hypotonic treatment with 0.056 M KCl was performed for 20 min, a fixation step and several washing steps using Carnoy’s fixative and final dropping of the suspension onto the slide’s surface.

### Murine metaphases from primary spleen

Mouse metaphase spreads were prepared from primary spleen and short-term culture with phytohemagglutinin. After 60 h of incubation at 37 °C/5 % CO_2_ the cells were harvested. Thirty minutes before harvesting the cells, colcemid (0.1 μg/ml final conc.) was added, followed by hypotonic treatment with 0.040 M KCl for 40 min, a fixation step and several washing steps using Carnoy’s fixative and final dropping of the suspension onto the slide’s surface.

### FISH methods

#### M-FISH

M-FISH (multiplex FISH using whole chromosome painting probes) was applied for the proper identification of homologous chromosomes in cross species-FISH applications on primates and mouse. For primates, a human M-FISH probe set based on glass needle dissected probes was used as described before [58]. An analog M-FISH probe set specific for mouse chromosomes was used to assign chromosomes in mouse metaphase spreads [59].

#### cenM-FISH

CenM-FISH (centromere multiplex FISH for all 24 human chromosomes) was applied for the proper identification of homologous chromosomes in poor quality metaphases of bone marrow preparations, according to previously published protocols [60].

#### pod-FISH

pod-FISH (parental origin determination FISH) was performed on metaphase spreads of a male proband and his parents (i.e. a family trio) derived from peripheral blood lymphocytes. Furthermore pod-FISH was applied on metaphase spreads of two clinical cases with karyotype mos 47,XY,+7/47,XY,+min(7),upd(7)mat isodisomy/46,XY,upd(7)mat isodisomy and 46,XY,upd(14)mat heterodisomy, respectively, derived from permanent EBV transformed cell lines. BAC clones for pod-FISH [19] were selected from CNV regions by http://projects.tcag.ca/variation/, the DNA was isolated, PCR amplified, and labeled by nick translation. Informative BAC clones that gave signal intensity differences on homologous chromosomes were further tested for parental origin. 10–25 metaphases were evaluated each. Signal differences not directly visible by eye were measured by software approaches like SCION or Axiovision software, Carl Zeiss MicroImaging GmbH, Germany.

## Additional files

Additional file 1: Figure S1. Inverted DAPI images from 9 metaphase spreads of the normal proband from the family trio exemplify the bilateral grouping of haploid chromosome sets. These metaphases were also subjected to further analysis by pod FISH for determining the parental origin of the homologous chromosomes in the proband. (TIF 1521 kb) Additional file 2: Figure S2. Bone marrow metaphases from an AML patient with mosaic trisomy 8 after cen-M-FISH. The karyotypes showing two or three times the chromosome 8, indicated with an arrow, depending on the parental location in blue (paternal) or red (maternal) haplogroups. (TIF 786 kb) Additional file 3: Figure S3. A) Inverted DAPI images from 3 metaphases of case 1 with 47,XY,+min(14) and maternal heterodisomy 14. Due to a maternal centromere polymorphism both chromosomes 14 can be distinguished by the size of the FISH signal in cenh + and cenh-. B) Inverted DAPI images from 3 metaphases of case 2 with 46,XY,upd(7)mat. C) Inverted DAPI images from 3 metaphases of case 3 with 47,XY,+inv dup(22)mat. The karyotypes are given red and blue labels to indicate the maternal and paternal haplotypes. Arrows indicate the additional minute chromosome. (TIF 826 kb) Additional file 4: Table S1. Primate samples used in this study. Abbreviation: PBL, peripheral blood lymphocyte. (DOCX 12 kb) Additional file 5: Figure S4. Metaphases from ten different primate species (see also Additional file 4: Table S1) demonstrating a genome-wise sorting of the haploid chromosome sets after M-FISH showing chromosome grouping and the closer location of homologous chromosomes next to the symmetry line. (TIF 727 kb) Additional file 6: Figure S5. Examples for observed mirror-image groups of chromosomes (left, labeled in same colors) and homologous chromosomes located next to each other along the symmetry line in HSA (A), MMU (B) and SSC (C). (TIF 359 kb) Additional file 7: Figure S6. Round shaped metaphase spread from Silvery Langur (TCR, *Trachypithecus cristata*). Measurement of DNA content by DAPI per area resulted in ^~^20 % independent if the whole area is counted or a pie slice reflecting a symmetric/round shaped distribution of DNA in the metaphase state of the cell cycle. (TIF 255 kb) Additional file 8: Figure S7. Metaphase plate from *Zellsubstanz, Kern und Zelltheilung* (1882) by Walther Flemming [13] (A) and chromosome “rosettes” from routine cytogenetic diagnostics in amniotic fluid cells after in situ culture (B) and a metaphase spread in “rosette” shape from peripheral blood lymphocytes (47,XX,+mar) after cenM-FISH (C). (TIF 876 kb)
