# Supplementary figures and images for: Chromosomes in a genome-wise order: evidence for metaphase architecture
Source: Mol Cytogenet. 2016 Apr 27;9:36. doi: 10.1186/s13039-016-0243-y (PMC4847357; doi:10.1186/s13039-016-0243-y)

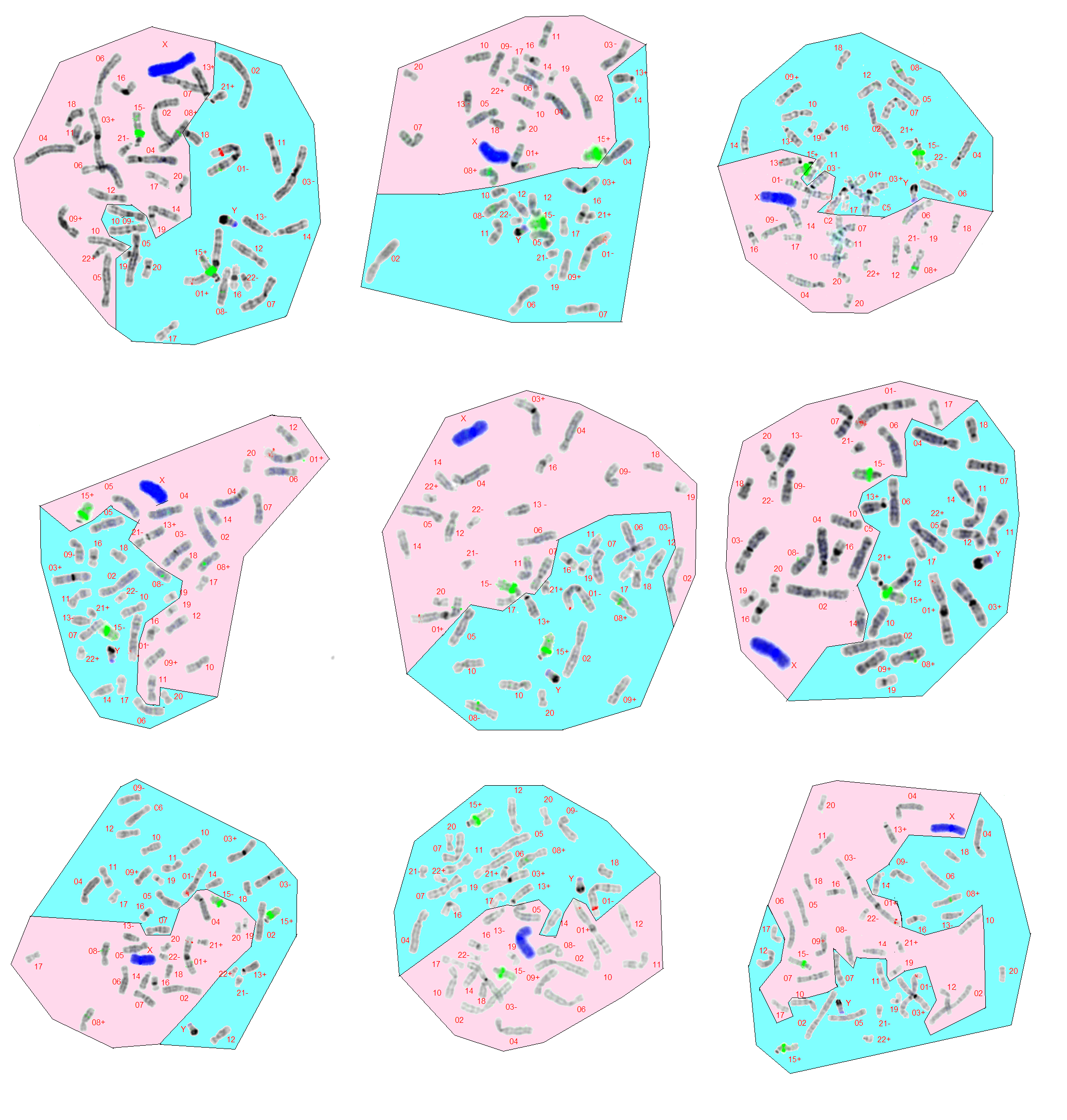

Supplement: Additional file 1: Figure S1. — Inverted DAPI images from 9 metaphase spreads of the normal proband from the family trio exemplify the bilateral grouping of haploid chromosome sets. These metaphases were also subjected to further analysis by pod FISH for determining the parental origin of the homologous chromosomes in the proband. (TIF 1521 kb) [file 13039_2016_243_MOESM1_ESM.tif]

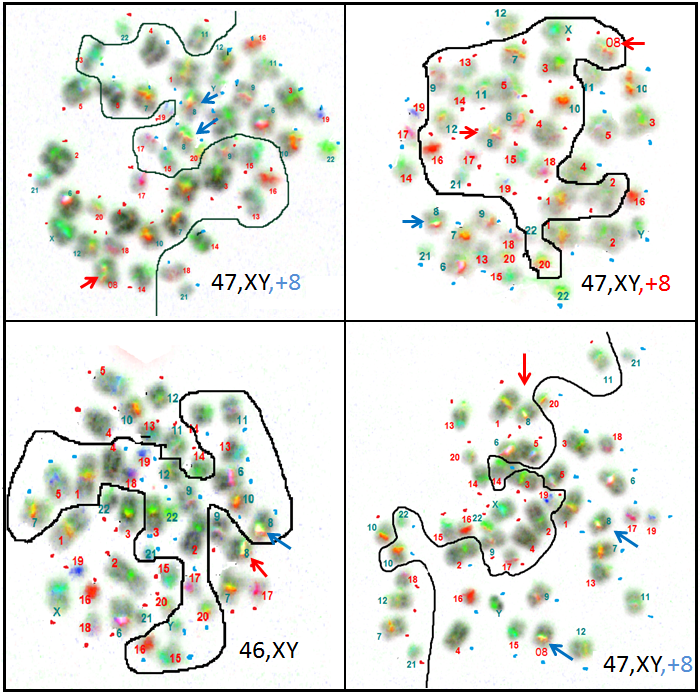

Supplement: Additional file 2: Figure S2. — Bone marrow metaphases from an AML patient with mosaic trisomy 8 after cen-M-FISH. The karyotypes showing two or three times the chromosome 8, indicated with an arrow, depending on the parental location in blue (paternal) or red (maternal) haplogroups. (TIF 786 kb) [file 13039_2016_243_MOESM2_ESM.tif]

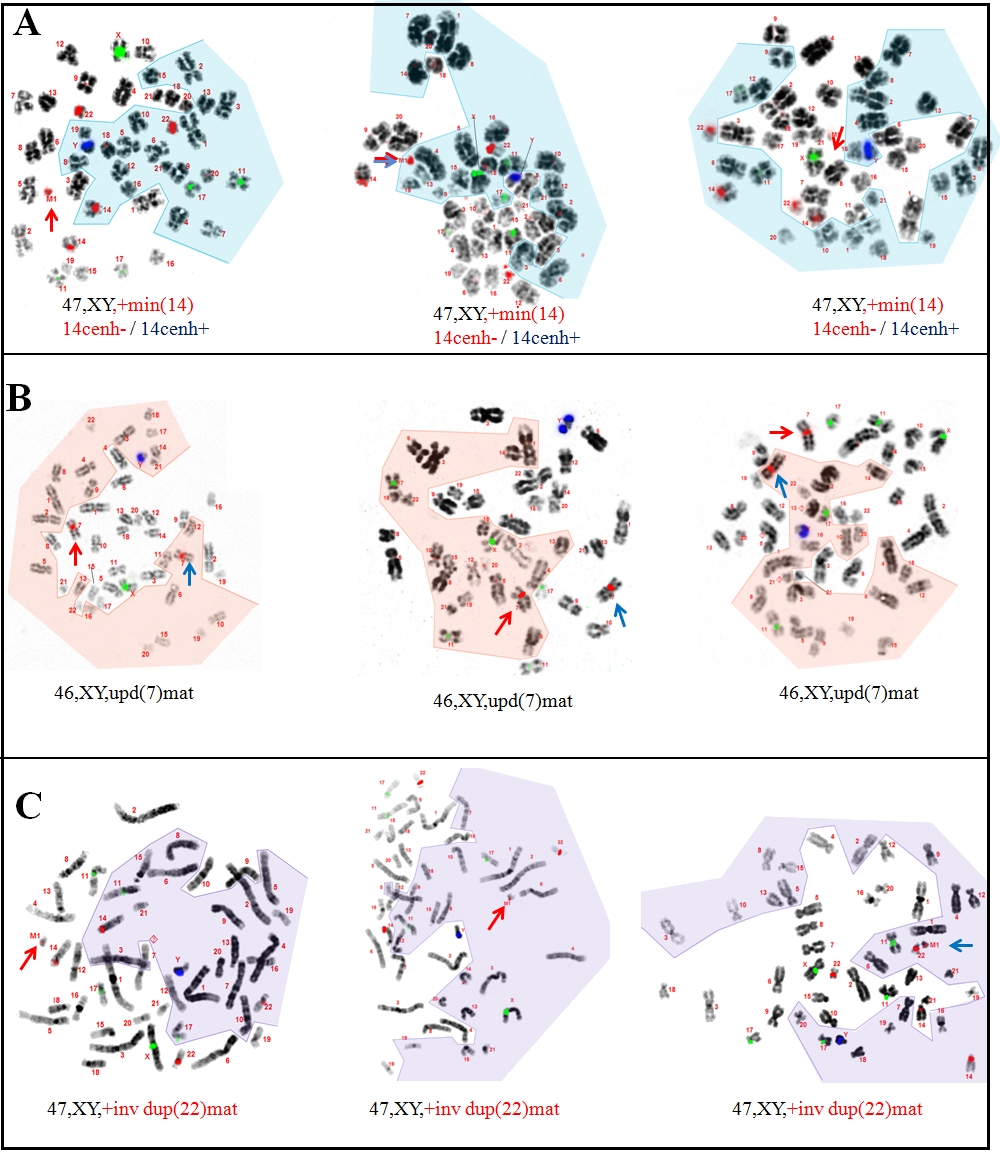

Supplement: Additional file 3: Figure S3. — A) Inverted DAPI images from 3 metaphases of case 1 with 47,XY,+min(14) and maternal heterodisomy 14. Due to a maternal centromere polymorphism both chromosomes 14 can be distinguished by the size of the FISH signal in cenh + and cenh-. B) Inverted DAPI images from 3 metaphases of case 2 with 46,XY,upd(7)mat. C) Inverted DAPI images from 3 metaphases of case 3 with 47,XY,+inv dup(22)mat. The karyotypes are given red and blue labels to indicate the maternal and paternal haplotypes. Arrows indicate the additional minute chromosome. (TIF 826 kb) [file 13039_2016_243_MOESM3_ESM.tif]

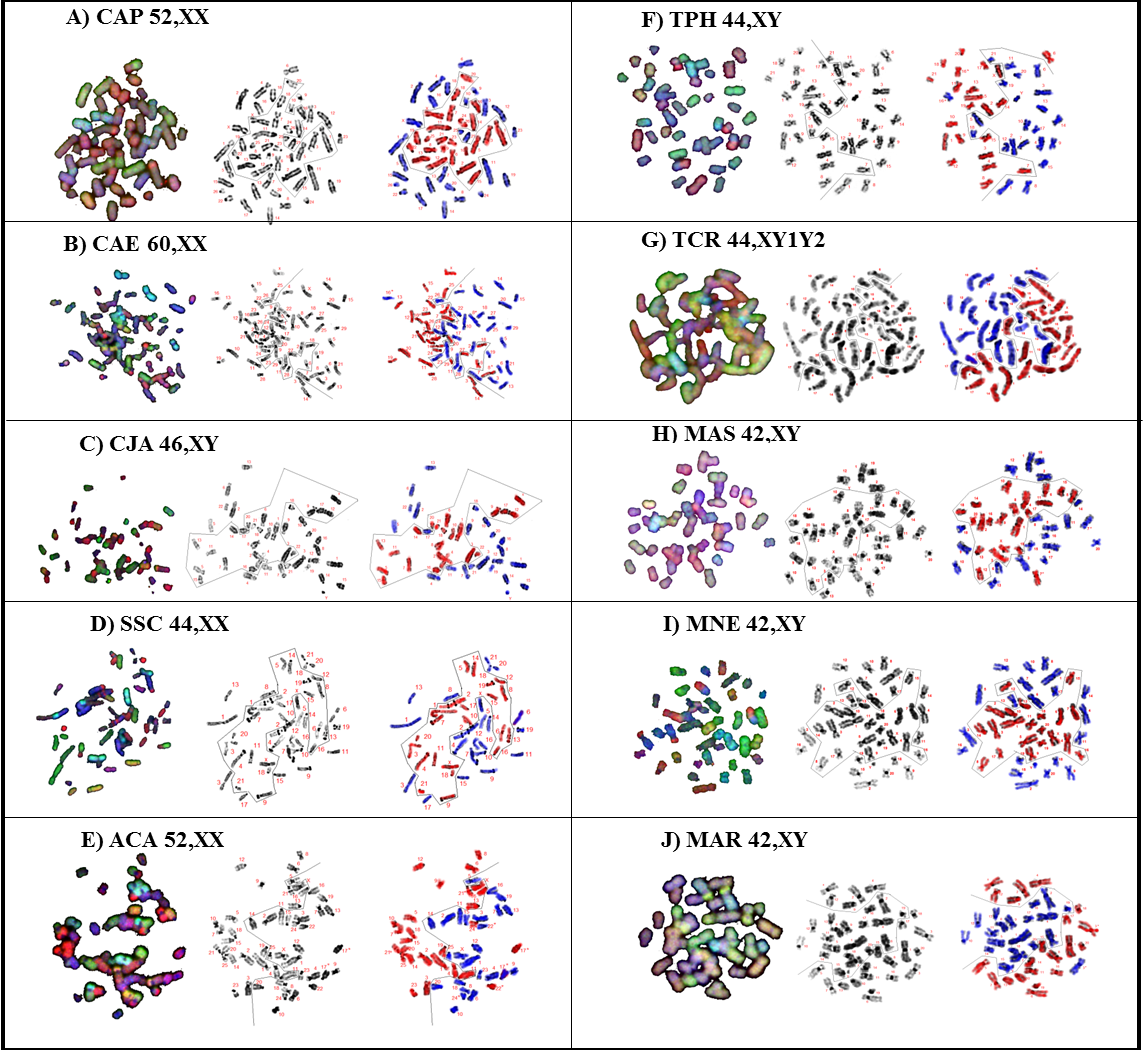

Supplement: Additional file 5: Figure S4. — Metaphases from ten different primate species (see also Additional file 4: Table S1) demonstrating a genome-wise sorting of the haploid chromosome sets after M-FISH showing chromosome grouping and the closer location of homologous chromosomes next to the symmetry line. (TIF 727 kb) [file 13039_2016_243_MOESM5_ESM.tif]

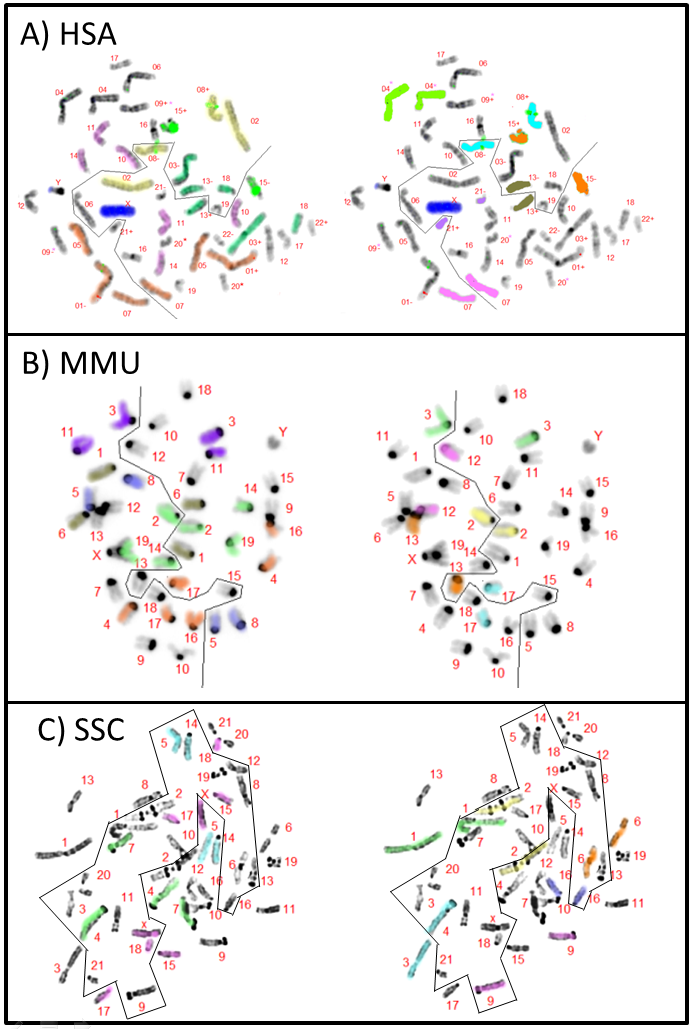

Supplement: Additional file 6: Figure S5. — Examples for observed mirror-image groups of chromosomes (left, labeled in same colors) and homologous chromosomes located next to each other along the symmetry line in HSA (A), MMU (B) and SSC (C). (TIF 359 kb) [file 13039_2016_243_MOESM6_ESM.tif]

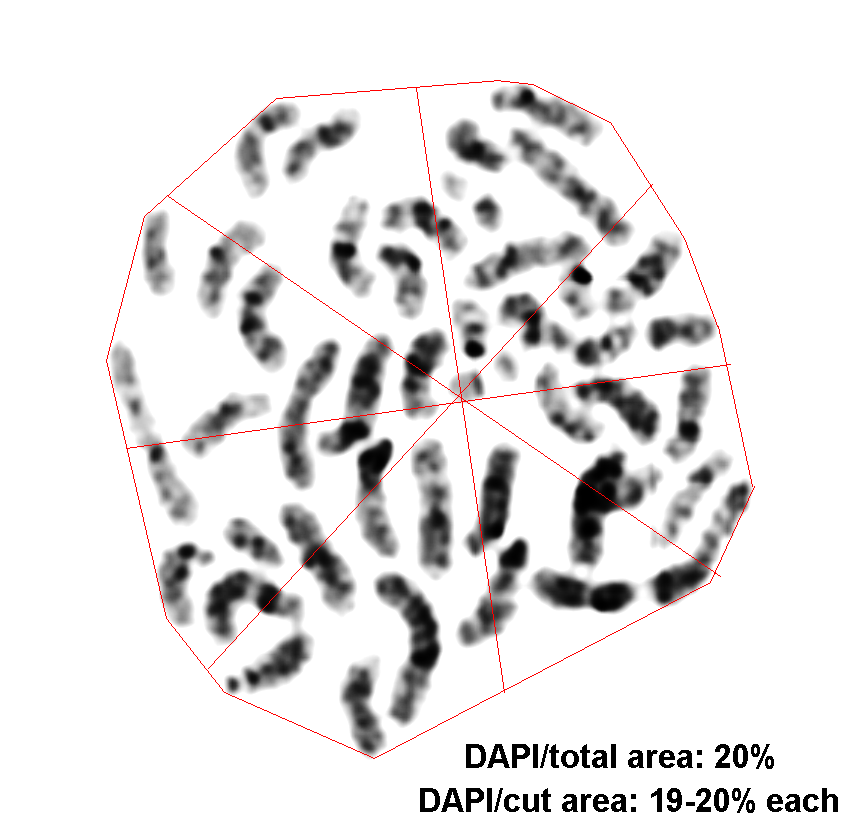

Supplement: Additional file 7: Figure S6. — Round shaped metaphase spread from Silvery Langur (TCR, Trachypithecus cristata). Measurement of DNA content by DAPI per area resulted in ~20 % independent if the whole area is counted or a pie slice reflecting a symmetric/round shaped distribution of DNA in the metaphase state of the cell cycle. (TIF 255 kb) [file 13039_2016_243_MOESM7_ESM.tif]

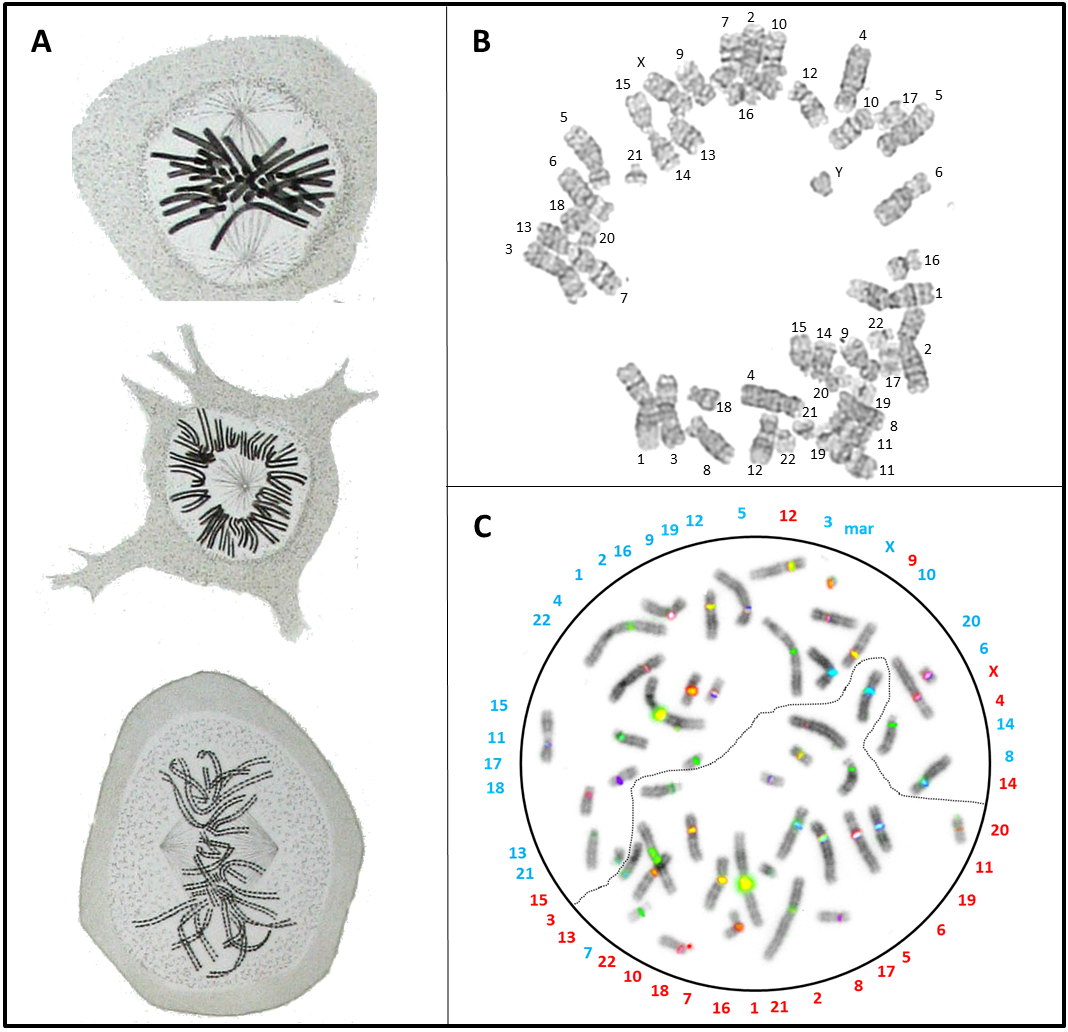

Supplement: Additional file 8: Figure S7. — Metaphase plate from Zellsubstanz, Kern und Zelltheilung (1882) by Walther Flemming [13] (A) and chromosome “rosettes” from routine cytogenetic diagnostics in amniotic fluid cells after in situ culture (B) and a metaphase spread in “rosette” shape from peripheral blood lymphocytes (47,XX,+mar) after cenM-FISH (C). (TIF 876 kb) [file 13039_2016_243_MOESM8_ESM.tif]
