# Supplementary material for: Chromosomes in a genome-wise order: evidence for metaphase architecture
Source: Mol Cytogenet. 2016 Apr 27;9:36. doi: 10.1186/s13039-016-0243-y (PMC4847357; doi:10.1186/s13039-016-0243-y)
Supplement: Additional file 4: Table S1. — Primate samples used in this study. Abbreviation: PBL, peripheral blood lymphocyte. (DOCX 12 kb) [file 13039_2016_243_MOESM4_ESM.docx]

Supplemental Table 1

| **Primate species** | **Karyotype** | **Origin** | **M-FISH example** |
| --- | --- | --- | --- |
| *Cebus apella*  *(CAP)* | *52,XX* | *lymphocytes*  *Brazil* | Fig 3A |
| *Chlorocebus aethiops (CAE)* | *60,XX* | *lymphocytes*  *Brazil* | Fig 3B |
| *Callithrix Jacchus-Sagui (CJA)* | *46,XY* | *lymphocytes*  *Brazil* | Fig 3C |
| *Saimiri Sciureus*  *(SSC)* | *44,XX* | *lymphocytes*  *Brazil* | Fig 3D |
| *Allouata Caraya*  *(ACA)* | *52,XX* | *lymphocytes*  *Brazil* | Fig 3E |
| *Trachypithecus phayrei (TPH)* | *44,XY* | *lymphocytes*  *Thailand* | Fig 3F |
| *Trachypithecus cristata (TCR)* | *44,XY1Y2* | *lymphocytes*  *Thailand* | Fig 3G |
| *Macaca assamensis*  *(MAS)* | *42,XY* | *lymphocytes*  *Thailand* | Fig 3H |
| *Macaca nemestrina*  *(MNE)* | *42,XY* | *lymphocytes*  *Thailand* | Fig 3I |
| *Macaca arctoides*  *(MAR)* | *42,XY* | *lymphocytes*  *Thailand* | Fig 3J |
